# The reconstruction on the game networks with binary-state and multi-state dynamics

**DOI:** 10.1371/journal.pone.0263939

**Published:** 2022-02-11

**Authors:** Junfang Wang, Jin-Li Guo

**Affiliations:** 1 Business school, University of Shanghai Science & Technology, Shanghai, China; 2 School of Mathematics & Statistics, North China University of water Resources & Electric Power, Zhengzhou, China; University of Electronic Science and Technology of China, CHINA

## Abstract

Reconstruction of network is to infer the relationship among nodes using observation data, which is helpful to reveal properties and functions of complex systems. In view of the low reconstruction accuracy based on small data and the subjectivity of threshold to infer adjacency matrix, the paper proposes two models: the quadratic compressive sensing (QCS) and integer compressive sensing (ICS). Then a combined method (CCS) is given based on QCS and ICS, which can be used on binary-state and multi-state dynamics. It is found that CCS is usually a superior method comparing with compressive sensing, LASSO on several networks with different structures and scales. And it can infer larger node correctly than the other two methods. The paper is conducive to reveal the hidden relationship with small data so that to understand, predicate and control a vast intricate system.

## 1 Introduction

Objects are often abstracted into complex networks in many social, economic, engineering and scientific fields, such as electric power networks, transportation networks, social networks, game networks, economic networks, protein interaction networks in biological systems, and so on. Many researches focused on network topology, network controlling and dynamic behavior of complex networks [1–5]. And yet mastering the structure networks is a prerequisite for understanding, predicating and controlling the system. Studies have shown that the information of adjacency among the group is often unknown and difficult to acquire directly [6], such as synaptic connections between neurons in the brain [7]. Therefore, it’s necessary to reconstruct it using other observable data, and we call it “dynamic network reconstruction”. Methods based on continuous and discrete dynamic systems have been proposed and applied one after another, such as Pearson or Spearman Correlation [8], Bayes [9,10], Mutual Information [11], Granger Causality method [12], Optimal Causation Entropy [13,14], Compressive sensing (CS) [15–19], LASSO [20], noise-driven [21,22], Maximum Likelihood [23–30], deep learning [31] and so on. And methods for dynamic network are also proposed [32]. More overviews about network reconstruction can be found in the literature [33–36]. Among them, CS is a method to solve the problems brought by high-dimensional data. For a sparse adjacency matrix, CS minimizes its *L*_1_ norm under the linear equations to reconstruct the adjacency matrix. It has been used in the fields of signal processing, numerical computation, computer vision, neuroscience, and so on.

During reconstructing, we maybe encounter the following questions. The first one, contradictory inferring, happened in the undirected and unweighted networks. To overcome the contradictory, Ma et al. [37] proposed the CBM basing on conflict frequency to solve the contradiction. Huang [2] proposed a compressed sensing model with constrain. Zhang et al. [38] identified large nodes through clustering and adjusting large nodes’ inferences via compressive sensing to solve conflict. Second, inferring the structures of nodes one by one demands extremely time for the large network. Shi et al. proposed an iteratively thresholded ridge regression screener for dimension reduction, and then employed the LASSO method to recover the network structure [39]. Another, if using the connecting probability to infer whether two nodes are adjacent, we must give a threshold, which is subjective. Moreover, the data we get are often insufficient for reconstructing the large network, especially on the large degree nodes. How to infer the network in a high precision with small data?

Here, two reconstruction models are proposed for game networks. They are compressive sensing model basing on 0−1 program (ICS) and compressive sensing model basing on quadratic program (QCS). We also give a combined method of ICS and QCS and discuss its performance. The main contribution of this article is as follows.

1. For evolutionary game systems, we propose an effective index to identify large nodes and network’s type.
2. Two reconstruction models, QCS and ICS, are proposed, and it needn’t to determine thresholds with ICS model.
3. A combined method for binary-state and multi-state systems is proposed, which has a higher performance, especially on multi-state dynamic. And the method is not limited to the reconstruction of the game network.

The rest of the article is organized as follows. In the section I, the paper gives the evolutionary game mechanism and evaluating indexes. In Section II, ICS and QCS models are given. Then, in Section III, the combined model of ICS and QCS is proposed on binary-state dynamics, and the performance of proposed method is compared with CS and LASSO. Section IV carries out a series of experiments to validate the performance of the proposed algorithm on multi-state dynamics including on some real networks. Finally, the conclusion and discussion remarks are given in Section V.

## 2. Game theory and reconstruction evaluation standard index

### 2.1 Evolutionary game theory

In a group, assuming individuals repeat the prisoner’s dilemma game in pairs, and they can adopt the strategies: C (unconditional cooperation), D (unconditional defection), ZD (zero-determinant strategy) [40], TFT (tit-for-tat) and WSLS (win stay, lost shift) [41] with a score matrix *A* between strategies as illustrated in Table 1.

**Table 1:** The scores for players X and Y in a single play of prisoner’s dilemma.

	C	ZD	D	WSLS	TFT
C	*b*−*c*	$\frac{\left(b^{2}-c^{2}\right)}{bx+c}$	−*c*	$\frac{b-2c}{2}$	*b*−*c*
ZD	$\frac{(b^{2}-c^{2})x}{bx+c}$	0	0	$\frac{(b^{2}-c^{2})x}{\left(1+2x)b+c(2+x\right)}$	0
D	*b*	0	0	$\frac{b}{2}$	0
WSLS	$\frac{2b-c}{2}$	$\frac{\left(b^{2}-c^{2}\right)}{\left(1+2x)b+c(2+x\right)}$	$-\frac{c}{2}$	*b*−*c*	$\frac{b-c}{2}$
TFT	*b*−*c*	0	0	$\frac{b-c}{2}$	$\frac{b-c}{2}$

In the round *τ*, the individual *i* plays games according to Table 1 with her immediate neighbors, and she obtains the accumulated payoff

$$U_{i}(\tau )=\sum_{j\in \Gamma (i)}u(i,j)$$ (1)

where *Γ*(*i*) is the neighbor set of individual *i*, and *u*(*i*,*j*) is the payoff of individual *i* from the game with neighbor *j*.

To optimize her behavior, her strategy *s*_*i*_ is replaced by the strategy of one randomly chosen neighbor, say *j* (with strategy *s*_*j*_), with the probability

$$P(s_{i}\leftarrow s_{j})=\frac{1}{1+e^{\left[(U_{i}(\tau )-U_{j}(\tau )/a\right]}}$$ (2)

where *a* represents her rational degree [42]. In the following, let *a* = 0.1.

### 2.2 Threshold model

The elements in the adjacency matrix should be binary, however the estimators we obtained will not be exactly 0 or 1. For node *i*, supposing that the adjacency values with other nodes are *k*_*j*_, *j* = 1,2,⋯*N*, *j*≠*i*. In a similar way to what done in Ref [26], we use a threshold value

$$\Delta_{i}=arg\;\underset{j}{\max}\left\{\frac{k_{j}}{k_{j+1}}(k_{j}-k_{j+1})\right\}$$ (3)

to separate the actual from the nonexistent links.

### 2.3 Three evaluation standard indexes

By comparing the inferring results to the true adjacency matrix, nodes can be classified into true positive (*TP*), false positive (*FP*), true negative (*TN*), or false negative (*FN*). To evaluate the reconstruction performance, we take three standard indexes: the area under the receiver operating characteristic curve (AUROC), the area under the precision-recall curve (AUPR) and success rate(*SR*)

$$SR=\sqrt{SREL*SRNC}$$ (4)

where $SREL=\frac{TP}{TP+FN},SRNC=TN/(FP+TN)$.

## 3 ICS and QCS models on game networks

For an undirected and unweighted network with *N* nodes, let *x*_*ij*_ = 1 if node *i* and *j* are connected, else *x*_*ij*_ = 0. Supposing that the strategy of each node is known in each round, then accumulated payoffs of node *i* from time period 1 to *M* can be expressed as

$$y(t)=\sum_{j\neq i}S_{ij}(t)x_{ij},t=1,2,\cdots ,M$$ (5)

where *S*_*ij*_(*t*) is known, which represents the score of the player *i* gained from playing with player *j* at round *t*. Let $Y=(y(1),y(2),\cdots ,y(M){)}^{T},X=(x_{i1},x_{i2},\cdots ,x_{i,i-1},x_{i,i+1},\cdots {,x}_{iN}{)}^{T},$ and matrix

$$F=(\begin{array}{cccccc} S_{i1}(1) & \cdots & S_{i,i-1}(1) & S_{i,i+1}(1) & \cdots & S_{iN}(1)\\ S_{i1}(2) & \cdots & S_{i,i-1}(2) & S_{i,i+1}(2) & \cdots & S_{iN}(2)\\ \cdots & & & & & \\ S_{i1}(M) & \cdots & S_{i,i-1}(M) & S_{i,i+1}(M) & \cdots & S_{iN}(M) \end{array})$$ (6)

For node *i*, the adjacent vector *X* satisfies the equation

Y=FX (7)

Since the elements in *X* are either 0 or 1, and *X* is a sparse vector, it can be solved by the following model (I), which minimize the number of non-zero elements in vector *X* under the constraints (7).

$$I:\;\left\{ \begin{array}{c} \min{\left\|X\right\|}_{0}\\ Y=FX\\ x_{ij}\in \{0,1\},j=1,2,\cdots ,N,j\neq i \end{array}\right.$$ (8)

where ‖X‖_0_ is the *L*_0_ norm of vector *X*.

On the other hand, as *x*(1−*x*) approaches 0, *x* approaches 0 or 1. If the elements of *X* are not limited to 0 and 1, we can minimize *x*_*ij*_(1−*x*_*ij*_) to make *x*_*ij*_ approach 0 or 1, which helps to determine the threshold too, so we propose the second reconstruction model

$$II:\;\left\{ \begin{array}{c} \min\sum_{j}x_{ij}(1-x_{ij})\\ Y=FX\\ 0\leq x_{ij}\leq 1,j=1,2,\cdots ,N,j\neq i \end{array}\right.$$ (9)

In model (**I**), the element *x*_*ij*_ is integer, so we call the model **ICS** (Integer Compressed Sensing). While in model (**II**), we minimize a quadratic function ∑*x*_*ij*_(1−*x*_*ij*_), thus we call it **QCS** (Quadratic Compressed Sensing). We can solve the model **I** and **II** with CPLEX and the function “quadprog” of MATLAB.

## 4. A combined compressive sensing model with binary-state dynamics

### 4.1 The influence of degree and sample size on reconstruction accuracy

To obtain the features of ICS and QCS, we analyze their performance from the perspective of node’s degree and sample size. Supposing that individuals play games on a scale-free network with 500 nodes and an average degree of 6 (unless noted, the following networks are similar), and their strategies are C and D strategy only. The payoffs between the strategies are listed in Table 1 with *b* = 1.5, *c* = 1. There are the following notes for the reconstruction.

**Fig 1:**
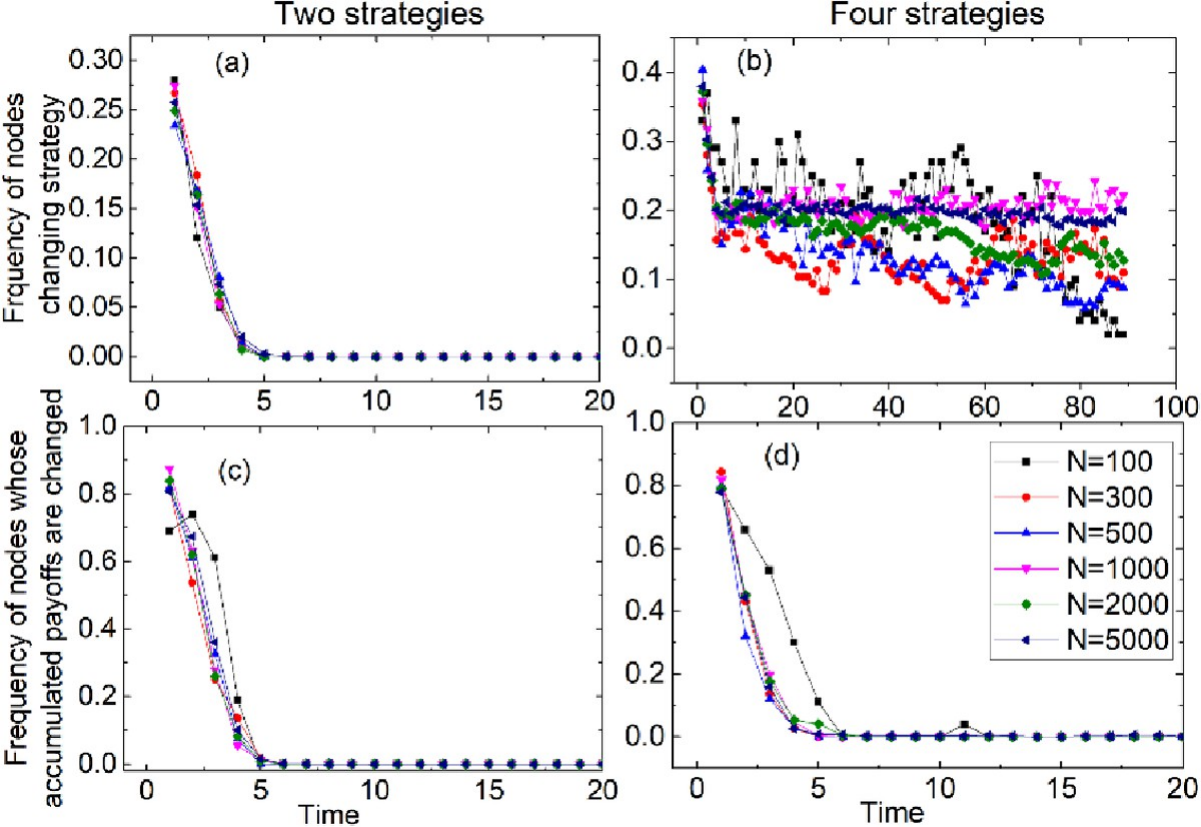
The frequencies of nodes who change strategies in each round in games with {C, D} strategy (Fig 1A) and {C, D, WSLS, ZD} strategy (Fig 1B). The frequencies of nodes whose accumulated payoffs are changed in each round in games with {C, D} strategy (Fig 1C) and {C, D, WSLS, ZD} strategy (Fig 1D).

(1) Considering that the game with strategies C and D approaches the steady state quickly and once it reaches the steady state, the data almost no longer change. In order to demonstrate such phenomenon, we simulate it on different scale networks. In each round, we count the frequency of nodes whose strategies are changed. The results are shown in Fig 1(A) and 1(C). It can be found that the strategies of all nodes are no longer changed after 4 rounds no matter the scale of networks, their payoffs as well. Furthermore, if there are four strategies in the games, although their strategies are not stable for a long time (see Fig 1(B)), yet the matrix *F* in Eq (7) is no longer changed. For example, the revenue of strategy D is always 0 no matter his co-player is D or ZD. Moreover, their payoffs also stabilize quickly (see Fig 1(D)). Hence, we make the nodes adopt strategy randomly for diverse data every 3 rounds.

(2) Since the feasible region of the ICS is discrete, it may take a long time to solve it. We will terminate the solution once the number of iteration exceeds 1×10^6^, and this node’s accuracy will be considered as 0.

(3) In the experiments, the nodes are sorted by the degree. The top 15% of the large nodes are grouped together, called **large group**, and the rest are the other group, called **small group**. It is worth noting that the division need not too strict.

Under the above agreements, the experiments are repeated 10 times. The results of ICS, QCS on two groups are shown in Table 2 with the samples size *M* (rounds of the games) from 10 to 40, where the evaluation index is average success rate of all nodes.

**Table 2:** Accuracy of QCS and ICS models on large and small group.

Sample size	ICS		QCS	
	Small	large	small	large
**10**	0.4283	0.4599	**0.5823**	**0.5464**
**15**	**0.7240**	0.5098	0.7722	**0.6043**
**20**	**0.9386**	0.6347	0.9061	**0.6603**
**25**	**0.9983**	**0.8041**	0.9614	0.7763
**30**	**1**	**0.9261**	0.9864	0.8489
**35**	**1**	**1**	0.9957	0.8879
**40**	**1**	**1**	1	0.9162

It can be found that QCS always has the highest accuracy no matter on large group or small group if data are scarce (10 samples), wherever ICS is always better than QCS if the samples are sufficient (more than 25 samples). Only if the sample size is 15 and 20 (insufficient), the results are complicated: the QCS is better on the large group, and ICS is better on small group. Therefore, if the sample is very scarce, it’s better to reconstruct with QCS on the whole network. If the sample is sufficient, we prefer use ICS. For the other case, we should combine them to infer the network: QCS for large nodes, ICS for small nodes.

From the above analysis, we should choose a proper model according to the sample size and the degree of node. Therefore, for the scale-free networks, we divide the sample size *M* into three levels according to its capacity: scarcity (*M*∈(0, *M*_1_)), insufficient (*M*∈[*M*_1_, *M*_2_]) and sufficient (*M*∈(*M*_2_, +∞)), wherever, for the networks of WS and ER, we divide the samples into two grades (*M*∈(0, *M*_3_)∪[*M*_3_, +∞)) since there are almost no large nodes in the networks. Next, we’ll analyze the threshold of sample size according to the type of the networks.

### 4.2 Judgment of sample capacity

Obviously, the threshold of sample size is related to the scale and density of the network. Seeing from Table 2, we can conclude that QCS is better than ICS on the majority of nodes if with scarcity samples. Hence define threshold

$$M_{1}=max\;\{M|\frac{\sum_{i=1}^{N}I({SR}_{ICS}(i)\leq {SR}_{QCS}(i))}{N}>0.7,with\;M\;samples\}$$ (10)

In the same way, let

$$M_{2}=min\;\{M|\frac{\sum_{i=1}^{N}I({SR}_{ICS}(i)\geq {SR}_{QCS}(i))}{N}>0.7,with\;M\;samples\}$$ (11)

where *I*(∙) is indicator function and *SR*(∙) is the success rate.

**Fig 2:**
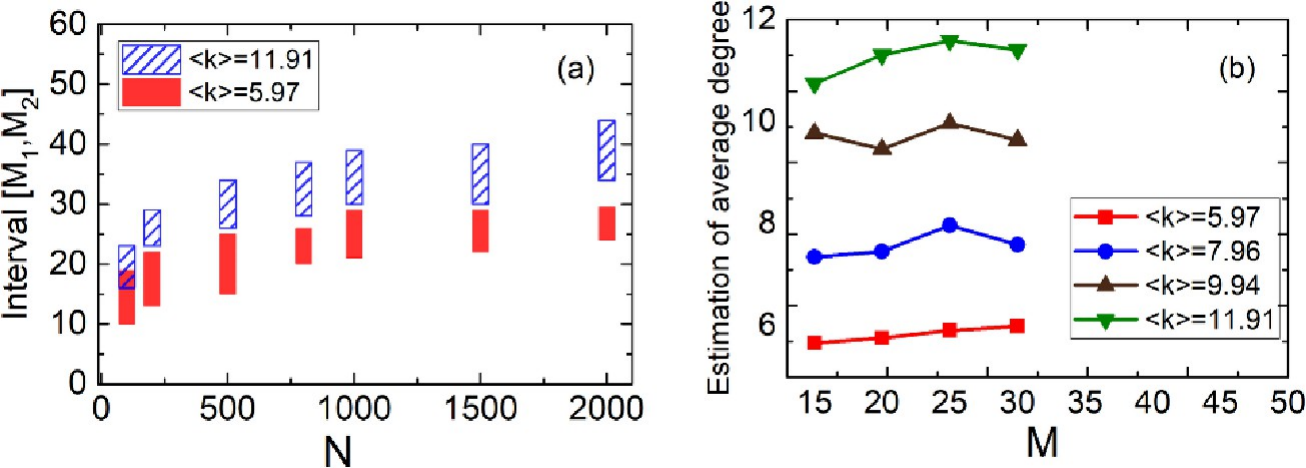
(a)The interval [*M*_1_, *M*_2_] on various networks. (b)The estimation of average degree with 500 nodes.

In order to find the regularity of the threshold, we simulate it on the scale-free networks with 100–3000 nodes and average degree 5.97–12. Some intervals of [*M*_1_, *M*_2_] have been shown in Fig 2(A). It can be found that intervals have strong statistical regularity. Hence, we establish polynomial regression models of *M*_1_ and *M*_2_ for *N* and average degree <*k*> with high fitness 0.924 and 0.925, respectively. They are

$${\hat{M}}_{1}=0.594+0.014N-2.875\times 10^{-6}N^{2}+1.479<K>$$ (12)

$${\hat{M}}_{2}=7.456+0.015N-3.438\times 10^{-6}N^{2}+1.585<K>$$ (13)

For the networks without large nodes, let

$${\hat{M}}_{3}=\frac{{\hat{M}}_{1}+{\hat{M}}_{2}}{2}$$ (14)

If the predicted value is a decimal, we take the smallest integer not less than it. We can utilize above equations to evaluate the samples so that to choose a proper reconstruction model, yet we also notice that it is necessary to acquire the average degree <*k*> of network with (Eqs 12 and 13).

In fact, the average degree of the network can be estimated with ICS alone. We can use the ICS model to make initial inference on the neighbors of each node so as to estimate the average degree of the whole network. We simulate it on the networks of 500 nodes with real average degrees 5.97, 7.96, 9.94,11.91 respectively (unknown), we estimate them with 15, 20, 25, 30 samples respectively. The results have been shown in Fig 2(B). We can see that the estimate fluctuates around the true value. Even though the estimate deviation reaches 1, it can be concluded from (Eqs 12 and 13) that the deviation of the thresholds about *M*_1_ *and M*_2_ is about 1.5 only, which hardly affects the evaluation of sample capacity.

Next, we’ll use above methods to predict *M*_1_ and *M*_2_ so that to judge the sample capacity on the scale-free network of 500,1000 nodes with 20 samples. The results are represented in Table 3. For the estimate of average degree is more accurate, the estimation of *M*_1_ is only 1 or 2 less than the real value, and *M*_2_ is more perfect with no difference from the real value on the network with 1000 nodes. It shows that (Eqs 12 and 13) are effective to estimate *M*_1_ *and M*_2_. In a word, we can evaluate the sample capacity through ICS and the number of nodes.

**Table 3:** The judgement of sample capacity (20 samples).

	*N* = 300 <*k*> = 5.96	*N* = 1000, <*k*> = 7.9
$\langle \hat{k}\rangle$	5.72	7.44
Estimation of [***M***_**1**_, ***M***_**2**_]	[13, 21]	[23, 31]
Real [***M***_**1**_, ***M***_**2**_]	[15, 22]	[24, 31]
Samples type	Insufficient	Scarcity

### 4.3 Identification of large nodes

For a scale-free networks, we should know which nodes are large nodes so as to choose proper model to reconstruct their neighbors if samples are insufficient (*M*∈[*M*_1_, *M*_2_]). How to identify the large nodes?

For an undirected network, inferring node’s neighbors one by one may lead to contradiction. CBM [37] believes that the accuracy of large node is lower than the small node with the method of CS, then the large node has a larger contradiction number. So CBM method identifies the large node through their contradiction number. For the game networks, we believe that the variance of node’s revenue sequence (*VR*) can also reflect its degree. It is because that node’s revenue will change as the neighbor’s strategy changes, and the large node has more neighbors, so her benefit fluctuation is larger than small node. For a node *i*, define

$$VR(i)=\frac{1}{M}\sum_{t=1}^{M}(y_{i}(t)-{{\bar{y}}_{i})}^{2}$$ (15)

where *M* is the length of revenue sequence, and ${\bar{y}}_{i}$ is the average revenue. We sort the *VR*s of all nodes and identify the large nodes with their order.

**Fig 3:**
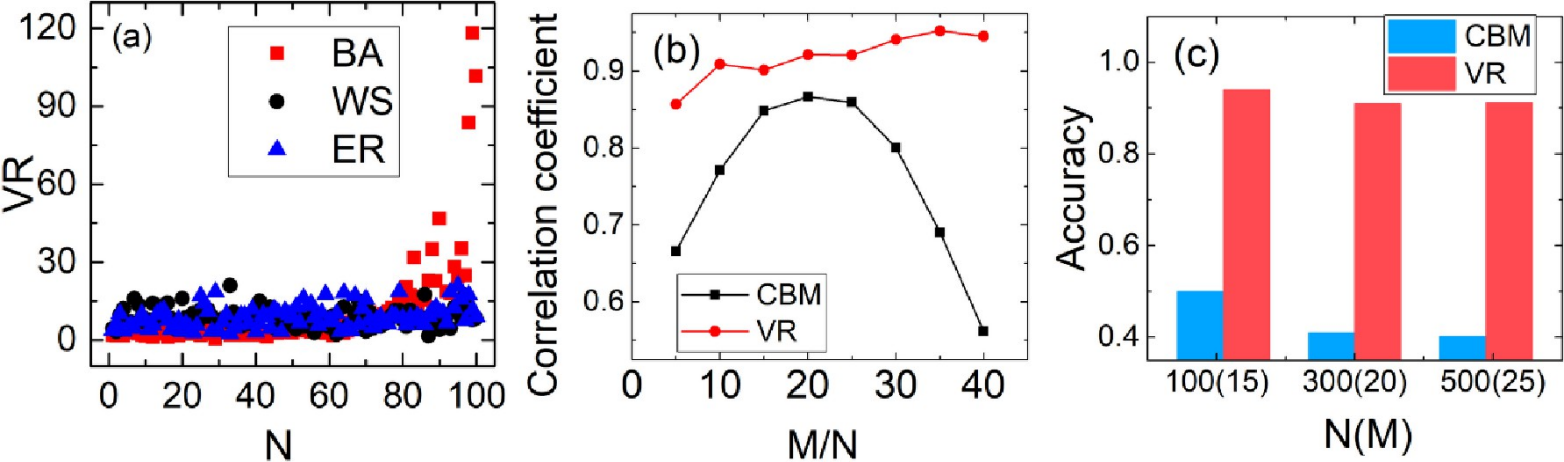
(a) The variance of each node’s payoff sequence (*VR*) on BA, WS and ER with 100 nodes and average degree 6, and the degree of the node on the right is larger than that on the left. (b) The correlation coefficient between *VR* (CBM) and the degree as the relative sample (M/N) increases. (c) The accuracy of identifying the top 10% large nodes with *VR* and CBM on the scale-free network with 100,300,500 nodes and 15, 20, 25 samples, respectively.

In the following, we simulate it on the network of BA, WS and ER with sample size 15 (insufficient samples). The *VR* of each node has been shown in Fig 3(A). We can see that *VR*s of the large nodes (at the right) are 20–100 times than that of the small nodes (at the left) in BA network, while the difference in other two networks is no obvious. Therefore, *VR* can be used to infer who are large nodes, in addition, we can evaluate the type of network with it.

Does the order of *VR* reflect the order of nodes’ degree really? We calculate the correlation coefficient between the *VR* and degree and compare it with the CBM. Fig 3(B) shows the results of two methods as the relative sample (M/N) increases. It is obvious that the correlation of *VR* is higher than that of CBM, and it is close to 1 as the sample size increases. Even if the sample size is only 5, the correlation coefficient is also more than 0.85, so *VR* can be used as an alternative indicator of the degree. We use it to identify the top 10% of large nodes on the scale-free network with 100,300,500 nodes withing sample size 15,20 and 25, respectively (insufficient in all cases). We do the same thing with CBM. The results are shown in Fig 3(C). The accuracy of *VR* is obviously higher than the CBM. It is no less than 0.9 in all cases, wherever CBM is no more than 0.5. It means that the *VR* is an effective index in identifying large nodes, which lays the foundation for the selection of model with insufficient data.

### 4.4 The combination compressive sensing model based on ICS and QCS

Basing on the features of ICS and QCS, we propose a combined model to reconstruct the whole network. The implementation steps are as follows:

Algorithm Proposed method for reconstruct the whole network based on evolutionary game data via ICS and QCS.

  **Input:**

  Strategy matrix *S* and accumulated payoffs matrix *Y* of each agent from time period 1 to *M*.

**Output:**

  The identification structure of the network.

  **Step 1:** For *i* = *1*:*N*

    Extract the revenue vector *Y*_*i*_ and *F*_*i*_ = *S*_*i*_*AS*_(*i*)_ of node *i*

    Using ICS model with *cplexbilp* package to estimate the neighbor set *x*_*0*_(*i*) of node *i* preliminarily.

    **End For**

    Calculating the average degree $\langle \hat{k}\rangle$ of the network.

  **Step 2: For** *i* = *1*:*N*

    $VR(i)=\frac{1}{M}\sum_{t=1}^{M}(Y_{i}(t)-{{\bar{Y}}_{i})}^{2}$

   **End For**

  Identify the type of network through the scatter plot of *VR*. If it is scale-free network, switch to Step 3, else to Step 4.

  **Step 3:**

   Taking $\langle \hat{k}\rangle$ and time period *M* into Eqs 12 and 13 to estimate *M*_*1*_ and *M*_*2*_. If sample size *M*<*M*_*1*_,switch to step 3.1, and if *M*>*M*_*2*_,switch to step 3.2, else to step 3.3.

   **Step 3.1:** Using QCS model with *quadprog* package to estimate the neighbors of each node.

   **Step 3.2:** Using ICS model with *cplexbilp* package to estimate the neighbors of each node if the number of iteration is no more than *1*×*10*^*6*^, otherwise with QCS.

   **Step 3.3:** After finding the large nodes according to the *VR* values, QCS are used to reconstruct their neighbors, while the other nodes are reconstructed using ICS (if the number of iteration is no more than *1*×*10*^*6*^).

  **Step 4:**

   Taking $\langle \hat{k}\rangle$ and *M* into Eqs 12 and 13 to estimate *M*_*3*_. If *M*<*M*_*3*_, switch to step 3.1, else to step 3.2.

**End**

Since the approach above combines ICS and QCS according to the scale of network and the sample size, it is called the combined compressed sensing method (CCS).

### 4.5 Reconstruction with CCS on the networks with different types and different sizes

Firstly, we simulate the performance of CCS and compare it with CS and LASSO on scale-free, small world and random networks, where the networks have the same size and same average degree. The results are shown in Table 4. It can be found that the accuracy of the CCS is almost the highest regardless of the type of the network, except the result on the scale-free network withing 10 samples. And CCS is always better than LASSO. It means that the advantages of CCS are hardly affected by the type of network.

**Table 4:** The success rate of three methods on different types of networks (*N* = 500, <*k*> = 6).

Sample size	scale-free network CS LASSO CCS			small network CS LASSO CCS			random network CS LASSO CCS		
**10**	**0.3976**	0.1642	0.3691	0.0709	0.0907	**0.5867**	0.3716	0.1073	**0.3961**
**15**	0.4352	0.2817	**0.4486**	0.2244	0.2203	**0.5911**	0.3859	0.2642	**0.4549**
**20**	0.5986	0.4533	**0.6555**	0.4151	0.3823	**0.6497**	0.5089	0.4161	**0.5039**
**25**	0.7529	0.6681	**0.7794**	0.6191	0.5533	**0.6716**	0.5968	0.5215	**0.6425**
**30**	0.8429	0.7926	**0.8992**	0.8072	0.7367	**0.9389**	0.7429	0.6771	**0.8773**
**35**	0.9110	0.8631	**0.9660**	0.9101	0.8360	**0.9970**	0.8548	0.8095	**0.9672**
**40**	0.9286	0.8937	**0.9740**	0.9811	0.9424	**1**	0.9234	0.8839	**0.9913**
**45**	0.9582	0.9365	**0.9792**	1	0.9919	**1**	0.9612	0.9257	**1**

**Fig 4:**
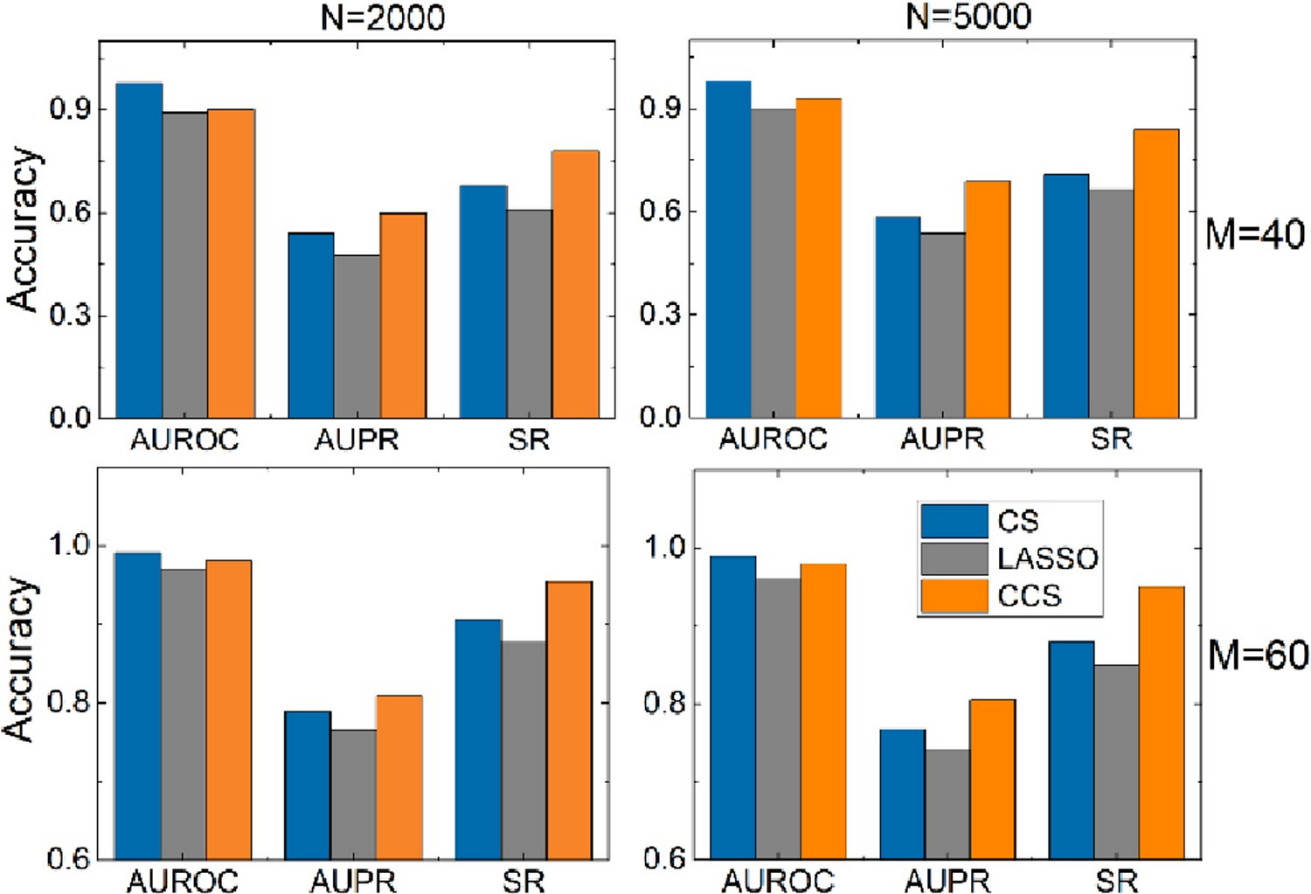
The performance of CCS, CS and LASSO on the scale-free networks with 2000, 5000 nodes, and sample sizes are 40, 60, respectively.

Next, we increase the scale to 2000 and 5000 with 40, 60 samples, respectively. The results are shown in Fig 4. It is not difficult to find that CCS has obvious advantages over CS in two (AUPR and SR) of the three evaluation indexes, and there isn’t much difference between CCS and CS in the third indicator (AUROC). Moreover, CCS is always better than LASSO on three indexes.

Synthesizing the simulations of the three methods on network with different types and different scales, we can conclude CCS is a superior method.

## 5 The combination compressive sensing model on multi-state dynamics

### 5.1 The influence of strategy to CCS model

In the following, the impact of game strategy and their number on reconstruction accuracy will be discussed. There are 5 strategies: C, D, TFT, WSLS and ZD. We design 8 groups with 5 strategies. The success rate (*SR*) on the scale-free network is shown in Table 5 withing 20 samples on the scale-free network with 500 nodes. We can find that the accuracy of CCS is always the best in three methods except the group of {C, TFT}. CCS is even 26–37% higher than the other two methods for the group (8). It means that the strategies in system play an important role on the CCS.

**Table 5:** The succeed rate (*SR*) of three methods under different strategy combinations.

	strategies	CCS	CS	LASSO	*NDP*	*MF*
**(1)**	C, TFT	0.1814	**0.2986**	0.2347	2	81.93
**(2)**	C, D	**0.4486**	0.4352	0.2817	4	54.88
**(3)**	C, D, ZD	**0.5889**	0.5435	0.4209	6	63.05
**(4)**	C, WSLS	**0.6141**	0.56	0.4787	3	54.71
**(5)**	C, D, TFT	**0.7342**	0.5775	0.4654	5	46.49
**(6)**	C, ZD	**0.7789**	0.5992	0.4481	4	30.30
**(7)**	C, D, WSLS	**0.9106**	0.6506	0.5493	8	30.21
**(8)**	C, D, ZD, WSLS	**0.92**	0.6807	0.5508	12	40.86

To find out the deep reasons for the high accuracy of CCS, we introduce two influencing factors: the number of different elements in payoff matrix (*NDP*) in each group, the maximum frequency of these element in the matrix *F* (*MF*). For example, in group (1), strategy C and strategy TFT take part in the games. There are only two different elements {*b*−*c*, (*b*−*c*)/2} in payoff matrix (see Table 1), and in the 10 experiments, the frequency of element *b*−*c* in matrix *F* is 81.93%. In the following, we’ll analyze how and why such two factors affect the accuracy of CCS through correlation and regression analysis of *SR* on *NDP* and *MF*. The results can be seen in Table 6.

**Table 6:** The multiple linear regression analysis of *SR* on *NDP* and *MF*.

Model			Unstandardized Coefficients	Standardized Coefficients	t	Sig.
*R* ^2^	0.933	*constant*	0.995		7.151	.001
*r*_*SR*,*NDP*_	0.743	*NDP*	0.030	0.382	2.815	.037
*r*_*SR*,*MF*_	-0.905	*MF*	-0.010	-0.711	5.237	.003

Firstly, the Pearson correlation coefficient between *SR* and *NDP* is 0.743, which means that the larger the *NDP*, the better the CCS. And the correlation coefficient between *MF* and *SR* is −0.905, which means there is a strong negative correlation between them. A regression equation is got with a high fitness (*R*^2^ = 0.933)

$$\hat{SR}=0.995+0.03NDP-0.01MF$$ (16)

And the influence on *SR* of *MF* is larger than *NDP* according to standardized coefficients (0.382, -0.711). The results show that *NDP* and *MF* of matrix *F* strongly affect the accuracy of CCS, especially *MF*. What do these two indicators mean? Why do they affect the accuracy of CCS?

As we all know the location identification of 0 and 1 in vector *X* decides the accuracy of CCS, and it depends on the diversity of matrix *F* in Eq (7). The more diverse the matrix *F*, the easier it is to identify their locations in vector *X* and the higher the reconstruction accuracy. If all elements are the same in matrix *F* extremely, the locations of 1s are not unique. In fact, the diversity of matrix *F* is reflected in two aspects: the number of different elements and the uniformity of the distribution of elements. The larger the number of different elements, the more uniform their distribution, the higher the accuracy of CCS. And if the maximum frequency is too high, which means that the elements are not uniform in matrix *F*, it will lead to a low precision.

Explain it in another way, the diversity of the matrix *F* depends on the number of strategies. The more the strategies, the better the CCS. For instance, the accuracy of the three-strategy groups (group 3,4,5,7) is higher than that of the two-strategy groups (group 1,2), at the same time it is lower than that of the four-strategy group (8). So multi-strategy game system is friendly to CCS, which is helpful for CCS to infer its structure.

Moreover, the diversity of the matrix *F* also depends on the strategies of the games. If one strategy makes other strategies disappear quickly, only one strategy left extremely, then the elements of matrix *F* are the same in many ranks. Available information from the data is little, so it is difficult to identify the true neighbors (all **1s** in vector *X*). Hence, the strategies, such as WSLS, ZD, which can protect the cooperators and coexist with other strategies are helpful to acquire diverse samples so as to reconstruct the network accurately. For example, unlike strategy D, which always eliminates strategy C, ZD strategy is a catalyst for cooperation. It makes the ratio of C and ZD more even and their payoffs are different, so it is more diverse in the group {C, ZD} than in group {C, D}. But in group {ZD, D, C}, as ZD and D get the same payoff 0 if they encounter, then the difference of elements’ frequency in this group is larger than that in the group {C, D}, but smaller than that in the group {C, ZD}. As a matter of course, *SR*_*C*,*D*_<*SR*_*C*,*D*,*ZD*_<*SR*_*C*,*ZD*_.

In summary, besides the number of initial strategies, the type also affects the performance of reconstruction directly.

### 5.2 The performance of CCS on scale-free network

To study the performance of CCS on the multi-strategy game network, we design the following two groups:

1. Three strategies {C, D, WSLS} in the games
2. Four strategies {C, D, WSLS, ZD} in the games.

**Fig 5:**
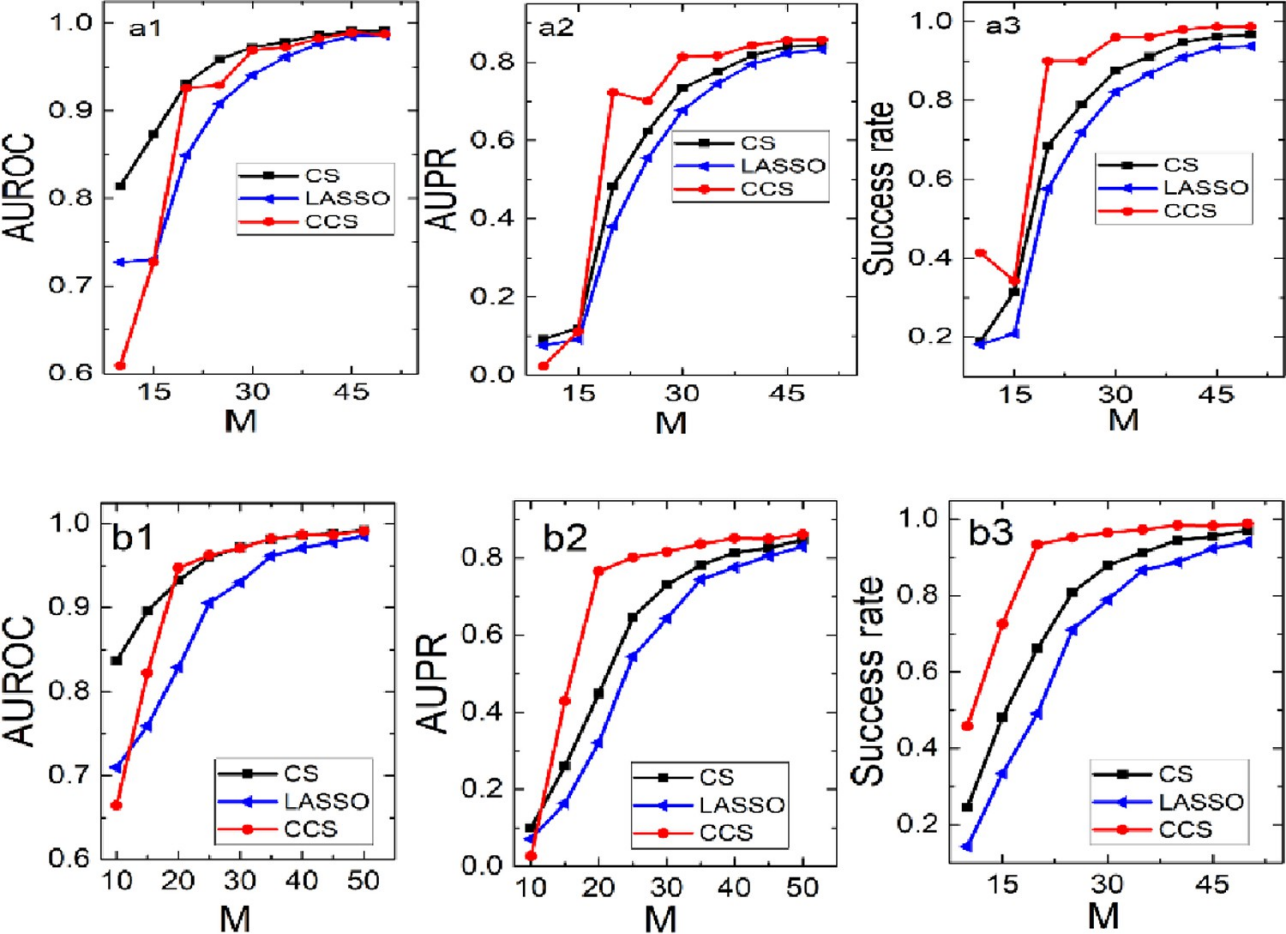
The performance of three methods as sample size increases from10 to 40. (a1-a3): Group {C, D, WSLS} (b1-b3): Group {C, D, WSLS, ZD}.

We compare the reconstruction performance of CCS, CS and LASSO in different sample sizes. The results are shown in Fig 5. It is obvious that CCS is almost the best with AUPR and success rate on both groups regardless of sample size. Although it is not the best on AUROC, its advantage gradually increases if ZD strategy involving (Fig 5.b1). As a whole, it stands out more when the samples are insufficient. For example, if *M* = 20, the success rate is more than 0.9 with CCS, however LASSO is only about 0.55, and CS is no more than 0.69. Moreover, the advantage of CCS is more obvious on four-strategy networks than on three-strategy networks.

### 5.3Noise environment experiments

In the real world, the practical environments are not as good as experimental environments. Limited by the accuracy and cost of observers, the observation data are not clean and pure, which contain a certain degree of noise. We test the performance of CCS in a noisy environment. Here, in the network with four strategies {C, D, WSLS, ZD} in the games, we assume that the observation noise is $\varepsilon ∼N(0,\sigma_{N}^{2})$, and *u*% of the samples are contaminated by noise. In Table 7, the variance of the noise is set as *σ*_*N*_ = 0.3, and *u* is set as 0.5, 1, respectively. The results demonstrate that the proposed method can cope with noise efficiently to a certain extent. The specific as follows:

**Table 7:** The success rate of CS, CCS, LASSO in a noisy environment.

*M*	*N* = 200, *u* = 0.5			*N* = 200, *u* = 1			*N* = 1000, *u* = 1		
	CS	CCS	LASSO	CS	CCS	LASSO	CS	CCS	LASSO
**10**	0.4015	**0.7315**	0.2761	0.4625	**0.6947**	0.2735	0.1224	**0.4057**	0.0846
**15**	0.7144	**0.8758**	0.5431	0.6273	**0.8057**	0.5055	0.3926	**0.6728**	0.2188
**20**	0.8141	**0.8736**	0.7181	0.7670	**0.8043**	0.7283	0.5220	**0.7297**	0.3962
**25**	0.8542	**0.8773**	0.8482	0.8004	0.7679	**0.8277**	0.5437	**0.7121**	0.4538
**30**	0.8472	0.8421	**0.9045**	0.7664	0.7383	**0.8726**	0.5913	**0.6993**	0.5387
**35**	0.8169	0.8221	**0.9166**	0.7170	0.7113	**0.9278**	0.6891	0.6708	**0.7947**

Firstly, although high rate of noise pollution is adverse to CCS, it still achieves the highest accuracy if the samples aren’t sufficient, as in the case of pure environment. Secondly, the bigger the network, the better for CCS. For example, increasing the scale of network from 200 to 1000, CCS can maintain its advantage in more cases (from *M*≤20 to *M*≤30). But it should be noticed that LASSO is more robust to the noise pollution if with large samples. Therefore, CCS is robust to noise in the case of small samples, and LASSO is robust in the case of large samples.

### 5.4 The results of CCS on real network

Compare with the generative small-world, random network and scale-free network, real networks may not have the obvious behavior characteristics; therefore, we choose 4 real networks to test the generalization of the proposed method: the football, the dolphin, the elegans and the social networks, including 115, 62, 453, 1858 nodes and average degrees 10.66, 5.13, 8.9 13.45, respectively.

#### 5.4.1 The macro-analysis of the reconstruction accuracy of the network

**Fig 6:**
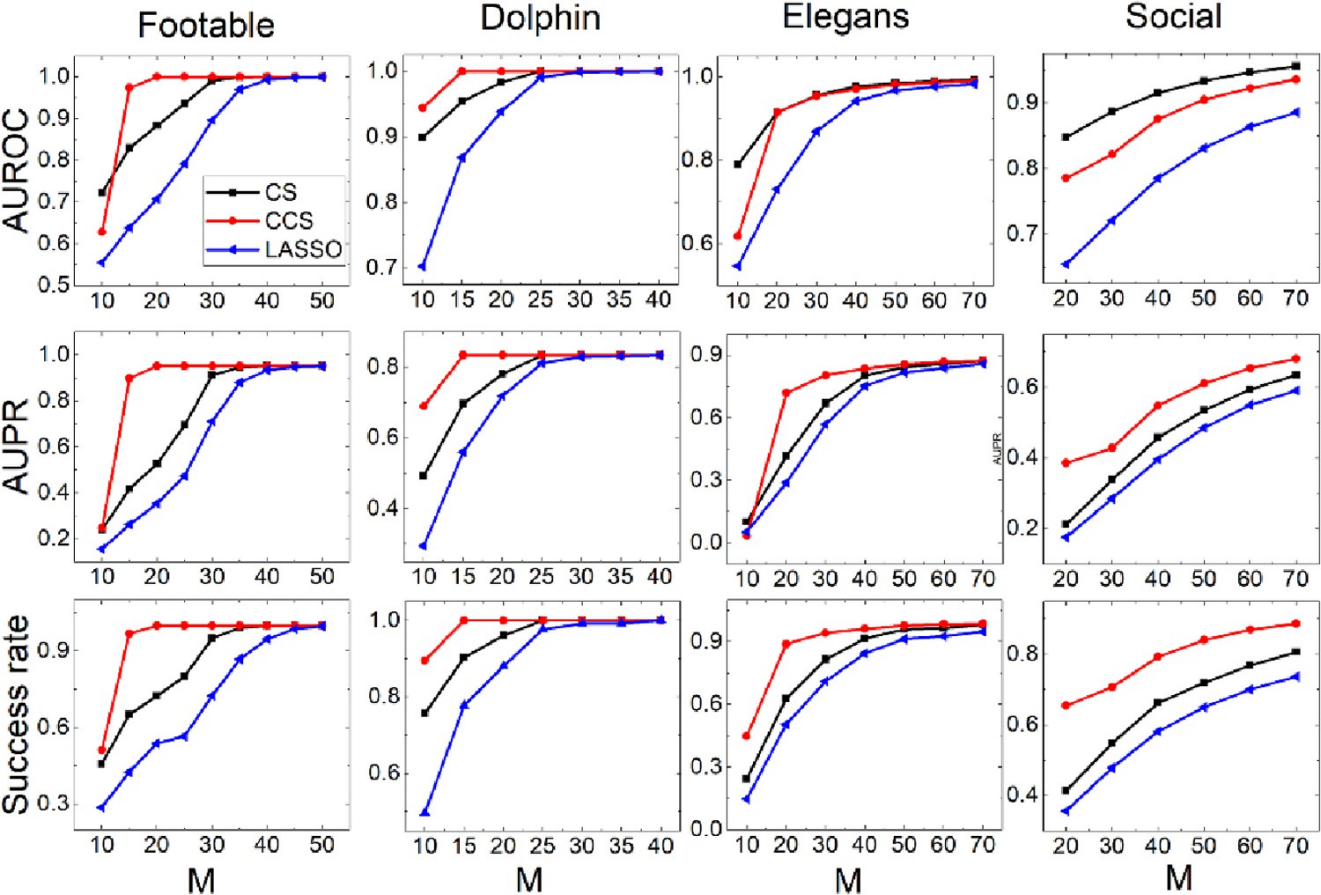
The performance of CCS, CS and LASSO is shown with three evaluation indexes on football, dolphin, elegans and social networks.

Suppose that the nodes can adopt four strategies: {C, D, ZD, WSLS}. The accuracy of three methods is shown in Fig 6. CCS always has the highest accuracy about AUPR and success rate, except for the occasional case with 10 samples. Furthermore, its reconstruction accuracy increases rapidly with the increase of samples, especially on the football network. It is worth mentioning that CCS is not always the best on AUROC. The sparser the network, the higher the reconstruction accuracy. It means that the sparse networks are better for CCS. It validates that CCS maintains similar referring features on real networks.

#### 5.4.2 The influence of node degree on completely correct frequency

**Fig 7:**
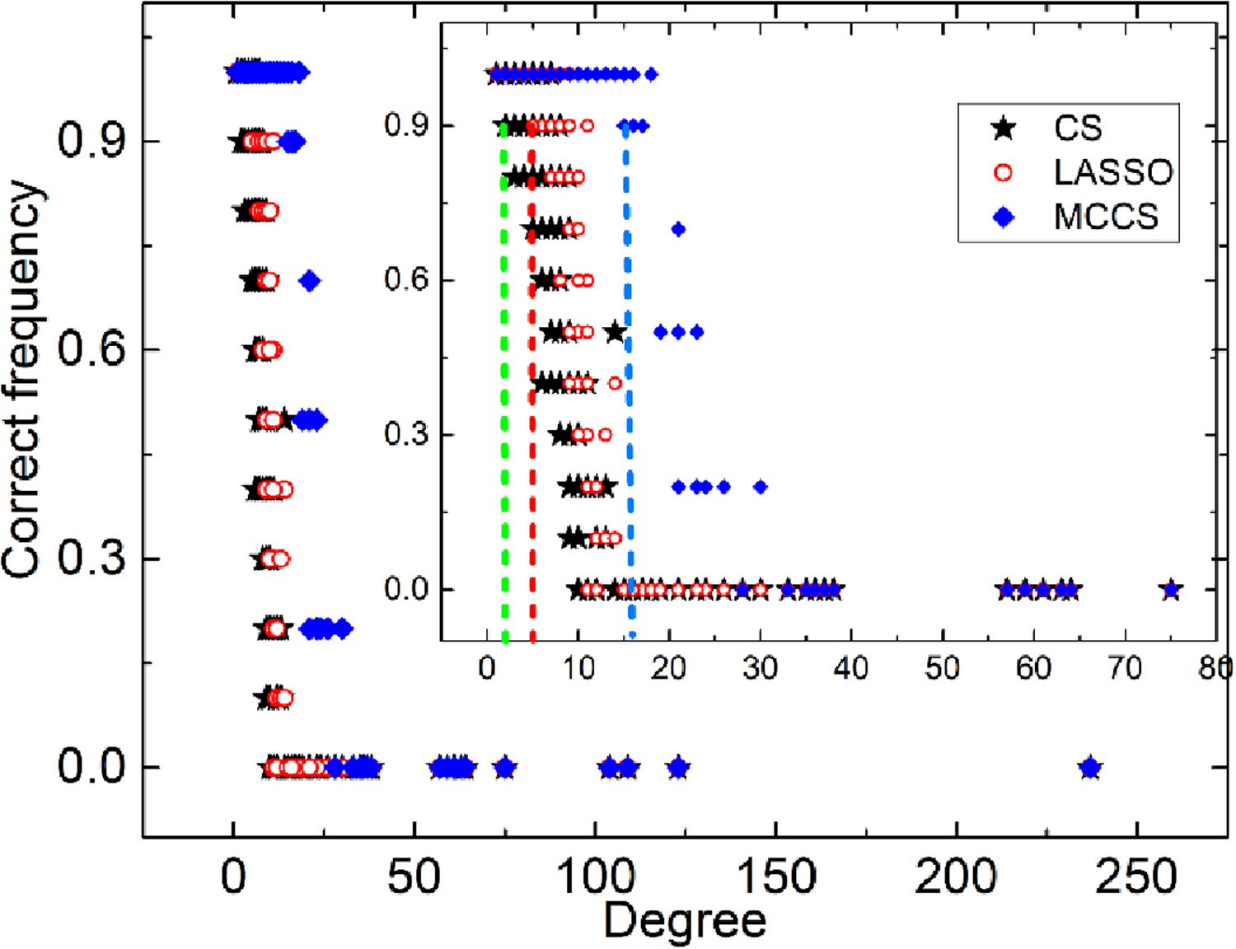
The completely correct frequency in 10 experiments on the elegans network under 50 samples.

As we know, not all nodes can be reconstructed correctly with insufficient samples. Which node is most likely to be reconstructed correctly? Is it related to its degree? Define completely correct frequency of node *i* as

$$fr(i)=\frac{n_{i}}{n}$$ (17)

where *n* is the number of experiments, and *n*_*i*_ is the frequency of completely correct reconstruction on node *i*. For example, if node *i* has a 100% success rate in 9 out of 10 experiments, then *fr*(*i*) = 0.9. In the following, three reconstruction methods are compared to analyze which method is most likely to be affected by the degree. Assuming that the samples size is 50, and each node is reconstructed 10 times. We calculate the completely correct frequency of each node and draw the scatter of completely correct frequency and degree in Fig 7.

Within expectation, the larger the degree, the lower the accuracy. The completely correct frequencies of all the large nodes are 0. To analyze the case of the smaller nodes, we zoom in the graph where degree is from 1 to 80. The difference among three methods can be found easily. Define the minimum degree of the nodes whose completely correct frequencies are less than 1

$$MD=min\{k_{i}|fr(i)<1,i=1,2,\cdots ,N\}$$ (18)

For the CCS, it is 16 (the degree on blue line). In other words, the nodes whose degree is no more than 15 can always be reconstructed correctly. In contrast, CS and LASSO are 2 and 5, respectively (green line and red line). It means that CCS can correctly reconstruct the larger nodes than CS and LASSO despite that all of them are not very friendly to large nodes. Therefore, CCS is more tolerant to the larger nodes.

#### 5.4.3 Coupled oscillations dynamics

In order to further verify the generality of the proposed structure identification method, the oscillator dynamics network is introduced and explored. Here, consider a complex Kuramoto model

$${\dot{\theta }}_{i}(\tau )=\omega_{i}+\sum_{j=1}^{N}a_{ij}sin\;(\theta_{j}-\theta_{i}),i=1,2,\cdots ,N$$ (19)

where *θ*_*i*_ and *ω*_*i*_ are the phase and the natural frequency of the ith oscillator. Assuming that

$$y_{i}={\dot{\theta }}_{i}-\omega_{i}$$

$$F_{i}={\left(sin\;(\theta_{j}-\theta_{i}\right)}_{j}$$

$$y_{i}=F_{i}A$$ (20)

**Fig 8:**
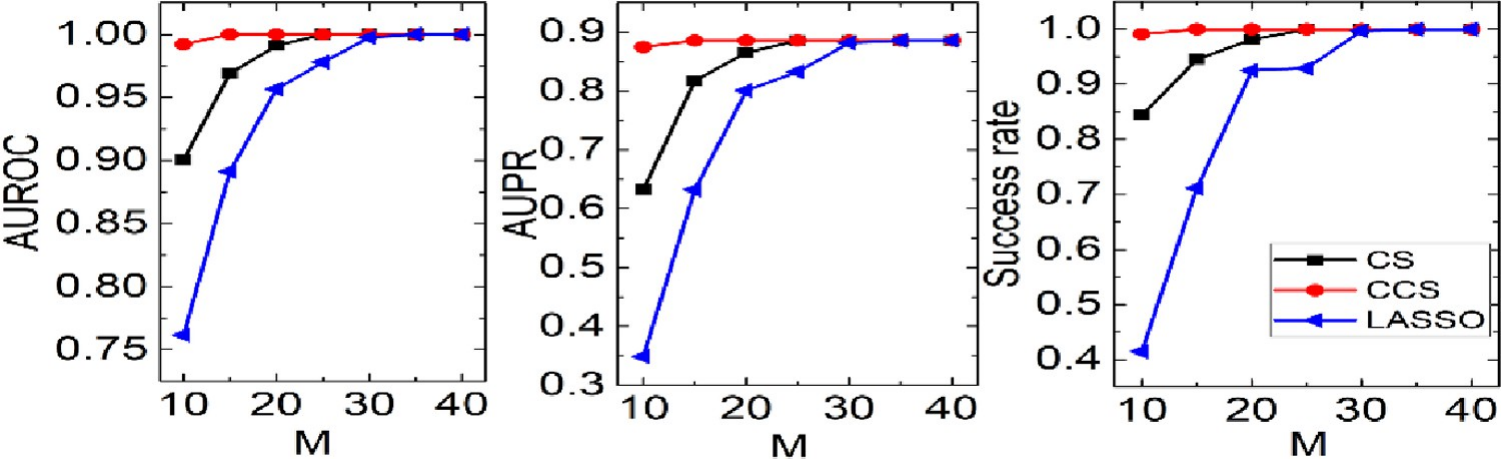
The performance of CCS, CS and LASSO is shown with three evaluation indexes on the coupled-oscillator dynamics. Network size *N* = 100, average degree <*k*> = 6.

Our target is to identify the network structure matrix *A* = (*a*_*ij*_). The identification results are shown in Fig 8. It is obvious that the proposed method is the best in three methods no matter what evaluation indexes are used. In particular, if there are only 10 observations, the success rate is near to 1 which is well above the other two methods. It means that CCS can also be used in other real systems besides evolutionary game systems.

## 6 Conclusions and discussions

In this paper, we propose two compressive sensing models: ICS and QCS firstly. According to the samples size, we also propose a combined model: CCS basing on ICS and QCS. It has been shown that the combined models usually have a higher accuracy compared with CS and LASSO on the networks with different types and different scales. In the multi-strategy system, it has an even better performance. The more strategies, the better performance. And the participation of strategies, which can improve group cooperation level, such as WSLS, ZD, etc., helpful for reconstruction with less data. In addition, CCS can correctly reconstruct the larger nodes than CS and LASSO. At the same time, it is robust under noise environment to a certain extent.

It is worth noting that the method is not limited to reconstruct the game networks although the paper discusses the reconstruction with evolutionary game data. As long as the linear constraint equation can be found from the system, the network can be reconstructed with our method. In addition, samples do not have to be time series data. It is worth mentioning that the combined compression sensing method should be improved in the following aspects. For example, sometimes we have to select model from ICS and QCS according to samples. Furthermore, sometimes we spend too long time on ICS. How to save time on ICS? They are worthy of attention in the future.

## Supporting information

S1 Appendix Football network. (MAT)

S2 Appendix Dolphin network. (TXT)

S3 Appendix Elegan network. (MAT)

S4 Appendix Social network. (XLSX)

S5 Appendix Code. (M)
